# NK Cells in HIV-1 Infection: From Basic Science to Vaccine Strategies

**DOI:** 10.3389/fimmu.2018.02290

**Published:** 2018-10-17

**Authors:** Lizdany Flórez-Álvarez, Juan C. Hernandez, Wildeman Zapata

**Affiliations:** ^1^Grupo Inmunovirología, Facultad de Medicina, Universidad de Antioquia UdeA, Medellín, Colombia; ^2^Infettare, Facultad de Medicina, Universidad Cooperativa de Colombia, Medellín, Colombia

**Keywords:** natural killer cells, HIV-1, HIV resistance, HIV vaccine, memory NK cells

## Abstract

NK cells play a key role in immune response against HIV infection. These cells can destroy infected cells and contribute to adequate and strong adaptive immune responses, by acting on dendritic, T, B, and even epithelial cells. Increased NK cell activity reflected by higher cytotoxic capacity, IFN-γ and chemokines (CCL3, CCL4, and CCL5) production, has been associated with resistance to HIV infection and delayed AIDS progression, demonstrating the importance of these cells in the antiviral response. Recently, a subpopulation of NK cells with adaptive characteristics has been described and associated with lower HIV viremia and control of infection. These evidences, together with some degree of protection shown in vaccine trials based on boosting NK cell activity, suggest that these cells can be a feasible option for new treatment and vaccination strategies to overcome limitations that, classical vaccination approaches, might have for this virus. This review is focus on the NK cells role during the immune response against HIV, including all the effector mechanisms associated to these cells; in addition, changes including phenotypic, functional and frequency modifications during HIV infection will be pointed, highlighting opportunities to vaccine development based in NK cells effector functions.

## Introduction

Natural killer (NK) cells play a key role in the host defense given their cytotoxic activity against tumors and virus-infected cells (1). In addition, they produce several cytokines including interferon gamma (IFN-γ), tumor necrosis factor alpha (TNF-α), granulocyte-macrophage colony-stimulating factor (GM-CSF) (2) and chemokines (macrophage inflammatory protein (MIP)-1α (CCL3), MIP-1β (CCL4) and CCL5, also known as RANTES (regulated on activation, normal T cell expressed and secreted) (3).

These cells, were named as natural killer cells because of their capacity to destroy tumor cells without prior sensitization (4). The identification of several donors with NK cell deficiencies, who were susceptible to severe and recurrent viral infections, provided evidence on the role of NK cells in the antiviral response, especially on herpes virus, hepatitis virus and poxvirus (5, 6). NK cells constitute about 10% of the mononuclear cells in human peripheral blood and their function are regulated by a number of germline-encoded activating/inhibitory receptors that together orchestrate their activation (7, 8).

These receptors can be divided into three groups, (i) killer immunoglobulin-like receptors (KIRs), (ii) natural cytotoxicity receptors (NCR) and (iii) NKG2/CD94 heterodimer family (C-type Lectin-like receptors). The KIRs include activating and inhibitory receptors that recognize major histocompatibility complex (MHC) class I associated with peptides, while NCRs a family of activating receptors, recognize viral derived products (9). NK cells are able to recognize host stress proteins up regulated during viral infections through C type Lectin-like receptor family (10). In addition, NK cell surveys the expression of MHC class I molecules through inhibitory receptors like NKG2A and KIRs to protect healthy cells from NK cell-mediated killing. NK cells also express Toll-like receptors (TLRs), and *in vitro* assays shows that TLR agonists can activate them, revealing their role in early defense against other pathogens than the virus (11).

In addition to the antiviral immune response, NK cells are implicated in tumor surveillance. Besides down regulation of HLA, NK cells can recognize several MHC-related ligands that are up-regulated on various tumors (12), including UL16-binding proteins (ULBP1-6) and MHC class I-chain-related proteins A and B (MICA and MICB) (13, 14). NK cells are also involved in regulatory functions, by improving CD8^+^ T cell responses against viral infection (15), inhibiting the size/functionality of the T cell response and regulating crosstalk network with dendritic cells (DCs) and neutrophils to promote or hamper the immune response (16, 17).

**Figure 1:**
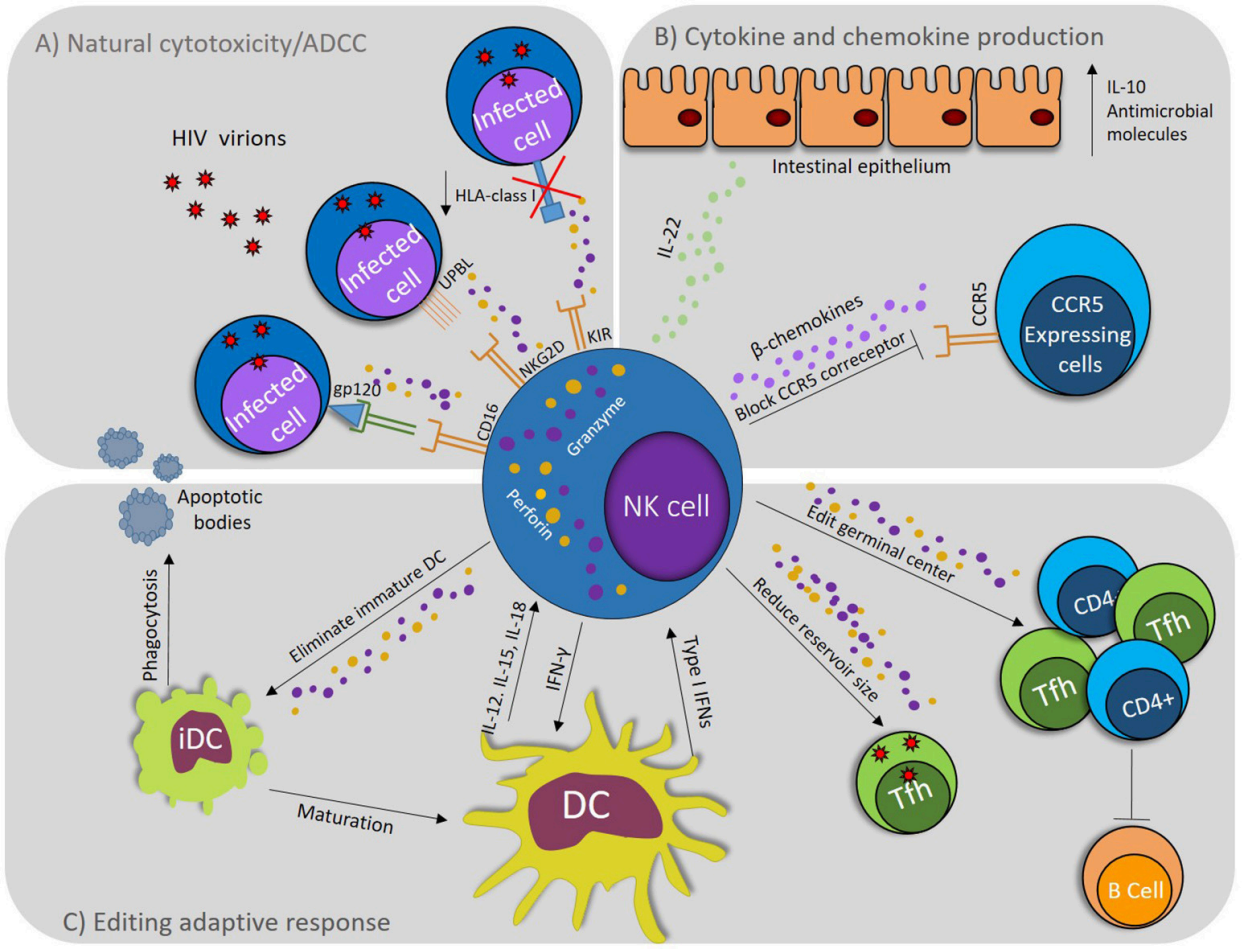
NK cell role during HIV-1 infection. **(A)** NK cells degranulate in response to activating signals via CD16 (FcγRIII), which binds Abs recognizing HIV proteins; also, by activating signals via NKG2D that binds stress signals like UPBL1, 2 and 3, which are up regulated on infected cells. Down regulation of HLA class I molecules induces activation by absence of inhibitory signals through KIR. **(B)** NK cells produce IL-22, which induce the production of antimicrobial molecules and IL-10 by epithelial cells. NK cells produce β-chemokines, which exert anti–HIV-1 activity *in vitro* by displacing the viral envelope glycoprotein gp120 from binding to CCR5 and by promoting CCR5 endocytosis. **(C)** iDCs uptake apoptotic bodies produced by NK cells activity inducing their maturation. NK cells realize DC editing eliminating iDCs to select mature DCs. DCs induce the activation of NK cells by producing IL-12, IL-18, and type I IFNs and NK cells produce IFN-γ inducing maturation of DCs. NK cells can eliminate CD4+ T cells and follicular helper T cells (Tfh), editing germinal center and affecting Abs production, but at the same time, by eliminating the Tfh, they reduce the HIV reservoirs.

The effector capacity of NK cells in the context of HIV-1 infection is not restricted to cytotoxic elimination of target cells. NK cells activation by the recognition of HIV-1-infected cells, may also lead to secretion of IFN-γ and MIP-1β, influencing the antiviral response and limiting viral spread (18). NK cells can also modulate adaptive response by a crosstalk with DCs (19), and shape the induction of antibodies through elimination of follicular T cells (Tfh) (20), demonstrating the multiple facets of NK cell in HIV-1 infection (Figure 1).

The antiviral response against HIV has been evaluated in different cohorts, that is the case of HIV controllers who maintain lower levels of HIV-1 replication in the absence of antiretroviral therapy, slow progressors and HIV-1-exposed seronegative individuals (HESN) who remain uninfected despite repeated exposure to the virus (21–23). Finding characteristics that explain their singularities, including an increased NK cell effector capacity, among other immune and genetic conditions, which opens a new field for HIV research with special attention in treatment and vaccination development, given the fall of classical approaches based on neutralizing antibodies.

This review will be focus on NK cells effector function during immune response against HIV infection, and the effect of this infection on NK cells number, phenotype and functionality highlighting the new field in HIV vaccine research based on NK cells.

## Effector functions of NK cells during HIV-1 infection

### Cytokine and chemokine production

Studies carried out in HESN cohorts, have shown that high levels of IFN-γ are associated with the seronegative status in uninfected infants born from HIV-1 infected mothers (HESN-infants) (24). Scott-Algara, et al. (25) reported an increased in IFN-γ and TNF-α production by NK cells from HIV-1-exposed seronegative intravenous drug users (HESN-IDU) compared with healthy controls (25). Similar results have been reported in different cohorts of serodiscordant couples (one partner is HIV negative and the other is HIV seropositive) (26).

A protective role of these cytokines could be explained by their ability to promote DCs maturation, up regulation of MHC molecules that favor antigen presentation and skew the adaptive response toward a Th1 profile, favoring the early control of HIV infection (27). However, these cytokines seem to have deleterious effects on chronically HIV-1 infected subjects. *in vitro* studies showed that TNF-α promotes HIV-1 gene expression via nuclear factor kappa B (NF-κB) pathway (28); in addition, there is a positive correlation between TNF-α levels and disease progression (29); this correlation may be due to HIV-1 capacity to induce TNF-α production through Tat, gp120 and Nef proteins, enhancing viral replication and inducing apoptosis of uninfected bystander cells, leading to immune escape by lacking recognition (30). Currently, the use of TNF-α inhibitors has been evaluated for the treatment of HIV-1 positive individuals, resulting in a reduction of the inflammatory state (31). IFN-γ is also involved in the chronic immune activation state that leads to immune exhaustion (32). These results show the differential role of these cytokines depending on the phase of infection.

NK cells produce large amounts of β-chemokines, CCL3, CCL4, and CCL5, natural ligands for CC-chemokine receptor 5 (CCR5), one of the coreceptors used by HIV to enter target cells. A study carried out on HIV-1-infected subjects showed that after stimulation with IL-2 and IL-15, the NK cells dramatically increased β-chemokines production, inhibiting viral entry to CD4^+^ T cells, limiting its spread (33). β-chemokines are also important in preventing mother to child transmission of HIV-1; studies developed with decidual NK cells from healthy pregnant woman, reported a partially inhibition of macrophages infection through CCL3, CCL4, and CCL5 production *in vitro* (18); this may be an explanation to the low transmission rate of HIV-1 from mother to child during the first trimester of pregnancy despite the permissiveness of the placenta and the decidual macrophages to be infected by HIV-1. PBMCs of elite controllers, showed specific *in vitro* resistance to R5 HIV strains while remaining susceptible to X4 strains, given higher levels of CCL3 compared with healthy donors (34). In addition, CCL3, CCL4, and CCL5 have been associated with natural resistance to HIV-1 infection in HESN-IDUs, who showed higher percentage of chemokines-producing NK cells, compared with seroconverted IDUs and healthy donors (25). Recently, genetic variants in CCL3 and CCL5 and their receptors have been associated with natural resistance to HIV-1 in a Colombian HESN cohort (35, 36).

Cella et al. (37) described an NK subpopulation named NK-22 with poor cytotoxic capacity and IFN-γ production, but a great ability to produce IL-22, IL-26 and leukemia inhibitor factor (LIF); cytokines that stimulate epithelial cells proliferation, expression of anti-apoptotic molecules and IL-10 (37). Currently, it is well known that these are innate lymphoid cells (ILC) which belongs to ILC3 group, while conventional NK cells belong to ILC1 group. However, there is evidence that conventional NK cells can also produce IL-22 (38–40) in order to regulate inflammation and immunity at the gastrointestinal tract and mucosal-associated lymphoid tissues (41). IL-22 is a member of the IL-10 family, which protects the epithelial cell barrier in the gut and other mucosal tissues of pathogens. IL-22 receptor is expressed almost exclusively on epithelial cells, where it initiates the STAT3 signaling pathway, inducing the production of antimicrobial molecules such as calcium-binding proteins of the S100 family, RegIII β, RegIII γ, lipocalin-2, IL-10 (42, 43), and β-defensins that have shown anti-HIV activity (44, 45).

Animal models of Simian immunodeficiency virus (SIV)/Simian-Human immunodeficiency virus (SHIV) have shown the association of IL-22 secretion in mucosa with microbial translocation. The amount of IL-22 secreted in mucosa was lower in the chronic phase than the early acute phase of SIV infection and the IL-22 levels were increased before microbial translocation occurred, suggesting that a decrease in IL-22 production favors microbial translocation (46).

Interestingly, transcriptome analysis carried out in HESN individuals have shown that IL-22 participates in the phenomenon of natural resistance to HIV. In this study, the expression of Granzyme B, Peroxiredoxin II (PRDX) and IL-22 was up-regulated in HESN compared with their HIV positive partners and healthy donors (47). Whereas PRDX is an enhancing factor for NK cells cytotoxicity to induce strong anti-HIV-1 activity, the IL-22 induce the production of acute phase serum proteins such as the serum amyloid A (SAA), a molecule that inhibits HIV-1 infection *in vitro*, modulating CXCR4 and CCR5 expression (47). This evidence suggests that NK cells promote a strong and well-directed adaptive anti-HIV response, including the modulation of HIV coreceptors. Besides promote the survival of epithelial cells in the gut mucosa, preventing microbial translocation, a key contributor to immune activation/exhaustion during HIV infection.

### Natural cytotoxicity and antibody-dependent cell-mediated cytotoxicity (ADCC)

Cytotoxicity is one of the main functions of NK cells; the binding of activating receptors with their ligands and the absence of inhibitory signals, results in NK cell activation and formation of lytic immunological synapse for polarized release of cytotoxic molecules stored in secretory lysosomes (48).

The effector ability of NK cells improves after immune licensing or education. In the immune licensing model, the acquisition of inhibitory KIRs specific for self HLA class I molecules, besides preventing auto-reactivity, is associated with the development of functional competence during maturation. Thus, exposure to target cells lacking HLA-I results in increased response rates of NK cells expressing inhibitory KIR for that missing HLA-I, whereas NK cells lacking self-inhibitory KIR remain hyporesponsive (49). On viral infections, down modulation of HLA molecules on infected cells can induce the activation of NK cells by absence of signals trough inhibitory KIR receptors. During HIV-1 infection the virus induces down modulation of HLA A/B expression to avoid recognition by cytotoxic lymphocytes (CTL) while leaving no classical HLA molecules unaltered, preventing NK cell activation (50).

Increased cytotoxicity in response to HLA down regulation has been associated with protection against HIV infection. Scott Algara et al. (25) reported that NK cell lytic activities against cell lines with reduced HLA expression (K562 and Daudi cells) were significantly increased in HESN-IDUs compared with either healthy donors or IDU who seroconverted during the study. In addition, HIV induces up regulation of specific ligands for NKG2D receptor, such as ULBP-1, 2, and 3 by Vpr accessory protein enhancing the susceptibility of HIV-1 infected cells to NK cell–mediated killing (51).

NK cells can also be activated by antibody-dependent stimuli in two ways; (i) NK cell activation dependent of antibodies (ADNKA) and (ii) Antibody-dependent cellular cytotoxicity (ADCC). Although both require the presence of opsonized target cells, ADNKA refers to NK cell activation measured by CD107a, IFN γ, and MIP1β expression; and ADCC point out the lysis of target cells by NK cells in the presence of an antibody bridge between NK cell and target cell, both are important in the context of HIV infection (52).

In this regard, several studies have demonstrated multiple antibody-dependent responses apart from neutralization that can be involved in protection against HIV infection and other viruses, these extra neutralizing functions include antibody-dependent compplement deposition, ADNKA, ADCC and antibody-mediated respiratory burst (53–55). A clinical trial for HIV/AIDS vaccine designed to evaluate non-neutralizing functions of vaccine-induced antibodies confirmed the correlation between HIV-specific antibodies and secretion of IFN-γ, CCL4 and expression of the degranulation marker CD107a on NK cells, from vaccine recipients (56). These extra-neutralizing antibody functions have been described in HIV elite controllers. Ackerman et al. (56) reported that spontaneous control of HIV infection showed by these subjects is related with polyfunctional antibody profile defined as the capacity to coordinately mediate ADCC and other NK antibody-dependent functions like IFN-γ secretion and maintained levels of neutralizing antibodies (57). These results suggest that HIV specific non-neutralizing antibodies, which mediate ADCC and ADNKA, may play a protective role against HIV infection, enhancing cytokine/chemokine production and lysis of target cells by NK cells, these mechanisms could be the base of several treatment and vaccine strategies for HIV infection, which will be mentioned further in this review.

Finally, given the effective activity of ADCC to eliminate infected cells, the HIV has developed strategies to evade this mechanism by inducing its dysfunction and limiting the presence of Env protein in the infected-cells surface through the accessory protein Vpu, which decrease the expression of tetherin, a cellular host restriction factor that tethers HIV virions on the cell surface, sequestering viral particles (58). Thus, decreased expression of tetherin reduces the ADCC, protecting infected cells from the NK cell activity (59).

### Editing the adaptive immune response

NK cells and DCs have a central role in antiviral immunity by modulate the adaptive immune response. Crosstalk between NK cells and DCs result in maturation of DC and in turn, DC cells up regulates NK cell effector function. This “co-activation” is given by cytokine production and cell to cell contact. IL-12 and IL-18 produced by activated conventional DCs promote IFN-γ production by NK cells, favoring Th1 response (60, 61), while plasmacitoid DCs (pDCs) produce type I IFN, which promotes NK cell proliferation and cytotoxicity. On the other hand, lysis of infected cells by NK cells provides a source of apoptotic bodies that immature DCs uptake, leading to their maturation and promoting viral antigen presentation to T cells (Figure 1) (61).

Mature DCs are the main antigen presenting cell (APCs) that initiate adaptive immune response, whereas, immature DCs are involved in tolerance and induction of regulatory T cells. *In vitro* models shown that NK/DC interaction results in lysis of immature DCs and preservation of mature DCs, in a process called “DC editing.” In this process, activated NK cells via NKp30 kill and secrete IFN-γ in response to immature DCs while mature DCs remain protected by up regulation of HLA molecules that induced inhibitory signals trough KIR receptors (62, 63). During HIV-1 infection, quantitative and qualitative alterations in DC and NK cells have been reported, affecting all these processes of reciprocal activation and preventing the development of adequate response (63).

*In vitro* studies have shown that HIV-1 accessory protein Nef has the capacity to affect NK/DC crosstalk. In presence of Nef-pulsed DCs, CD56^bright^ cells exhibit high capacity to produce IFN-γ, while CD56^dim^ showed a reduction in the cytotoxic capacity. Likewise, NK cells showed a significant reduction of IL-10, GM-CSF, MIP-1α, and RANTES secretion but increased TNF-α production, resulting in increased viral replication (19).

In addition, NK cells can directly modulate T and B cell responses. Immunoglobulin class switching and generation of memory by T cells can be enhanced by the cytotoxic activity and IFN-γ production of NK cells (64, 65). At the same time, studies carried out in mouse models have shown that in early moments of infection, NK cells can inhibit the generation of long-lived specific memory T and B cells, as well as specific antibodies by eliminating CD4^+^ activated T cells and Tfh. In mouse models, the NK cells depletion result in higher numbers of CD4^+^ and CD8^+^ memory T cells with polyfunctional profile during acute infection (20). In addition, (20) reported that in absence of NK cells, the infected mouse shown higher proportion of Tfh cells during acute infection and better formation of germinal centers (GC), which was reflected in higher numbers of antibody-secreting cells compared with NK cells sufficient mouse who shown reduced frequencies antibody secreting cells, even for months after infection (20).

The elimination of Tfh cells by NK cell affects the GC formation preventing the establishment of humoral response during acute phase of infection; however, it has been reported that elimination of Tfh cell during HIV infection could be beneficial, because these cells support HIV-1 replication in viremic individuals (66); therefore, NK cells may limit the size of the reservoir that is established after the acute phase of infection. This phenomenon has been described in animal models of SIV were natural hostess (animals that do not progress to disease despite presenting high viremias) like african green monkeys shown an enhanced capacity to control viral replication in lymph nodes compared with non-natural hostess like macaques that progress to disease. This capacity was associated with higher number of NK cells present in the lymph node (LN), specifically into the B-cell follicle of african green monkeys, this pattern of migration was associated with higher levels of CXCR5 expression on NK cells from african green monkeyscompared with macaques (67). The capacity of natural SIV hostess to control viral replication in LN has been related with a better control of inflammation, absence of Tfh infection and preservation of LN architecture and function that are some of the principal differences between pathogenic and non-pathogenic SIV/HIV infection (68).

This evidence suggests the important role of NK cells in different phases of HIV infection and offers a plausible target for immune modulation to avoid the elimination of cells forming part of GC in early moments of HIV infection, which could increase the antibodies production but promoting elimination of reservoirs in chronic infection. The beneficial effect of NK cells on adaptive immunity depends in great manner on the context, highlighting the need for addressing the activity of NK cells in a careful way during HIV infection. For instance, during curative intervention, targeting Tfh cells could lead to reduce the reservoir, but after vaccination is necessary to disable their suppressive effects to promote adequate humoral responses (69). Therefore, the impact of NK cells interventions, which could have a repercussion on adaptive immune response development, offers a new alternative in the design of methodologies for HIV prevention and cure.

## NK cell phenotype and frequencies during HIV-1 infection

NK cells have been classified in two major subsets based on the surface expression of CD56 (neural cell adhesion molecule, N-CAM). CD56^bright^ NK cells represent around 10% of peripheral blood NK cells, these cells are CD16^neg^ and express CD25 (High affinity IL-2R), whereas CD56^dim^ NK cells (around 90%) are CD16^pos^ and express CD122/132 (intermediate affinity IL-2R) (70, 71); differences in IL-2 receptor expression are reflected in their proliferative capacity.

Differences between these two subsets also include homing molecules and effector capacity. CD56^bright^ express CCR7 and CD62L, allowing them to migrate to secondary lymphoid organs; in fact, they are the most common NK cells in this tissue (70). CD56^bright^ are also characterized by low presence of lytic granules and the production of high amounts of IFN-γ, TNF-α, and GM-CSF (72). In contrast, CD56^dim^ preferentially migrate to inflamed peripheral tissues by CXCR1, CX3CR1, and ChemR23 and they express higher amounts of lytic granules. CD56^dim^ NK cells are considered the mature stage of NK cells; these cells come from a differentiation process involving loss of inhibitory receptors, like NKG2A and gain of CD16, KIR, and CD57 receptors (73). Despite the vast majority of NK cells can be included in these two groups, another subpopulation have been defined, CD56^−^ NK cells that express high density of CD16 molecules; these cells are NK cells with low cytotoxic activity and cytokines production; this functional altered subpopulation increase during chronic viral infections like HIV-1 (74).

Several changes in NK cell receptor repertoire and functionality are observed during HIV infection, some of them have been related with progression but others are still under study. Recently, it has been observed that treatment-naïve HIV-infected individuals showed a significant loss of the CCR7^+^/CD56^bright^ population, this change is associated with higher viral loads (75). Studies carried out in HIV and hepatitis C virus (HCV) coinfected individuals, showed that CD27^+^/ CD56^bright^ NK cells can help to clear of acute HCV infection by IFN-γ-mediated suppression, thus the alterations in CD56^bright^ subset can be related, not only with the loss of HIV control, but also with decreased capacity to eliminate other pathogens (76).

The loss of CD56^dim^ NK cell subset during HIV-1 infection has been also observed, especially in less differentiated CD56^dim^ cells, such as CD57^−^ and CD57^dim^ subpopulations (77, 78). The loss of CD56^+^ NK cells is associated with expansion of CD56^neg^/CD16^+^ NK cell population; this aberrant population expresses low levels of NKp30 and NKp46, reduced IFN-γ production, impaired cytotoxicity and poor ADCC response (79); in contrast, they produce significant amounts of MIP-1β (80).

Additional to the reduction in the CD56 expression, the expression of immunoregulatory molecules such as Siglec-7 and Tim-3 are also altered during HIV-1 infection. Siglec-7 is an adhesion inhibitory receptor, preferentially expressed by mature NK cells. Siglec-7^+^ NK cells displayed higher levels of activating receptors, increased CD107a expression and IFN-γ production than Siglec-7^−^ NK cells (81). Siglec-7 is considered one of the early surface markers down regulated in HIV-1 infection, associated with the expansion of dysfunctional NK cell subsets. Siglec-7 expression is suppressed throughout the course of infection, and its down regulation is associated with higher viremia (82). On the other hand, NK cells expressing high amounts of Tim-3 are fully responsive by cytokine production and cytotoxicity; this molecule is also an exhaustion marker (83). Tim-3 is also reduced in HIV-1 infected individuals; moreover, these individuals also showed high plasma levels of Gal-9, a Tim-3 ligand (84). Persistent Gal-9 stimuli via Tim-3, might result in loss of functionality and accumulation of NK Tim-3^low^ population, this might by an explanation to NK cell dysfunction observed in chronic HIV-1 infection (84).

Animal models of SIV shown accumulation of CD56^−^CD16^+^ NK cells in lymph nodes of infected animals compared with naïve animals. NK cells from lymph nodes demonstrated an enhanced cytotoxic capacity in acute infection models. Furthermore, Tim-3 was up regulated on NK cells from lymph nodes in chronically infected animals (85), suggesting that NK cells present a cytotoxic phenotype during acute SIV infection but may become dysfunctional and exhausted in chronic phase of disease. In addition to the alteration of NK cells in peripheral blood and lymphoid organs, a depletion of NK-22 cells in mucosa of SIV-infected macaques also has been reported. The loss of this population was associated with increased intestinal mucosal damage and microbial translocation (46, 86, 87), both implicated in immune hyperactivation and immune exhaustion, which are characteristics of the final stage of HIV infection.

## NK cell repertoire is associated with HIV progression and resistance

The study of long term non-progressors, controllers and HESN has revealed the existence of mechanisms for HIV resistance. NK cells are part of these mechanisms, exhibiting increased cytotoxicity and higher production of soluble mediators that have been associated with the expression of certain NK cells phenotypes.

The presence of the activator receptor KIR3DS1 on NK cells in combination with HLA-Bw4-I80, is one of the most reported phenotype associated with delayed AIDS progression and resistance to infection (32, 88–90). NK cells expressing KIR3DS1 are licensed by this receptor to activate in response to HLA-Bw4 expressed on target cells and they show an improved capacity of both cytotoxicity and IFN-γ production, apparently by HLA-Bw4 recognition in a peptide-dependent manner (91). However, the expression of KIR3DS1 in absence of HLA-Bw4-I80 allele has been associated with rapid progression to AIDS (92), probably because licensed NK cells through this KIR receptor are less responsive to target cells expressing another HLA allele, according with the immune licensing model.

The differential expression of the inhibitory lectin type C receptor, NKG2A, also has been related with an improved response against HIV-infected cells. NKG2A^+^ NK cells exhibit higher response against HLA null cells, and infected CD4+ T cells compared to NKG2A^−^ NK cells. In addition, these cells exhibit a poly-functional profile with co-expression of CD107a, IFN-γ, and CCL4, indicating that NKG2A receptors may have a role in the anti-HIV responses mediated by NK cells (93).

In 2006, a phenotype of NK cells with “memory” characteristics was described. O'Leary et al. showed that *rag*^−/−^ mouse can be sensitized against hapten. In this model, NK cells mediated an antigen-specific long-lived immunological recall response called contact hypersensitivity reaction for at least 4 weeks (94).

**Figure 2:**
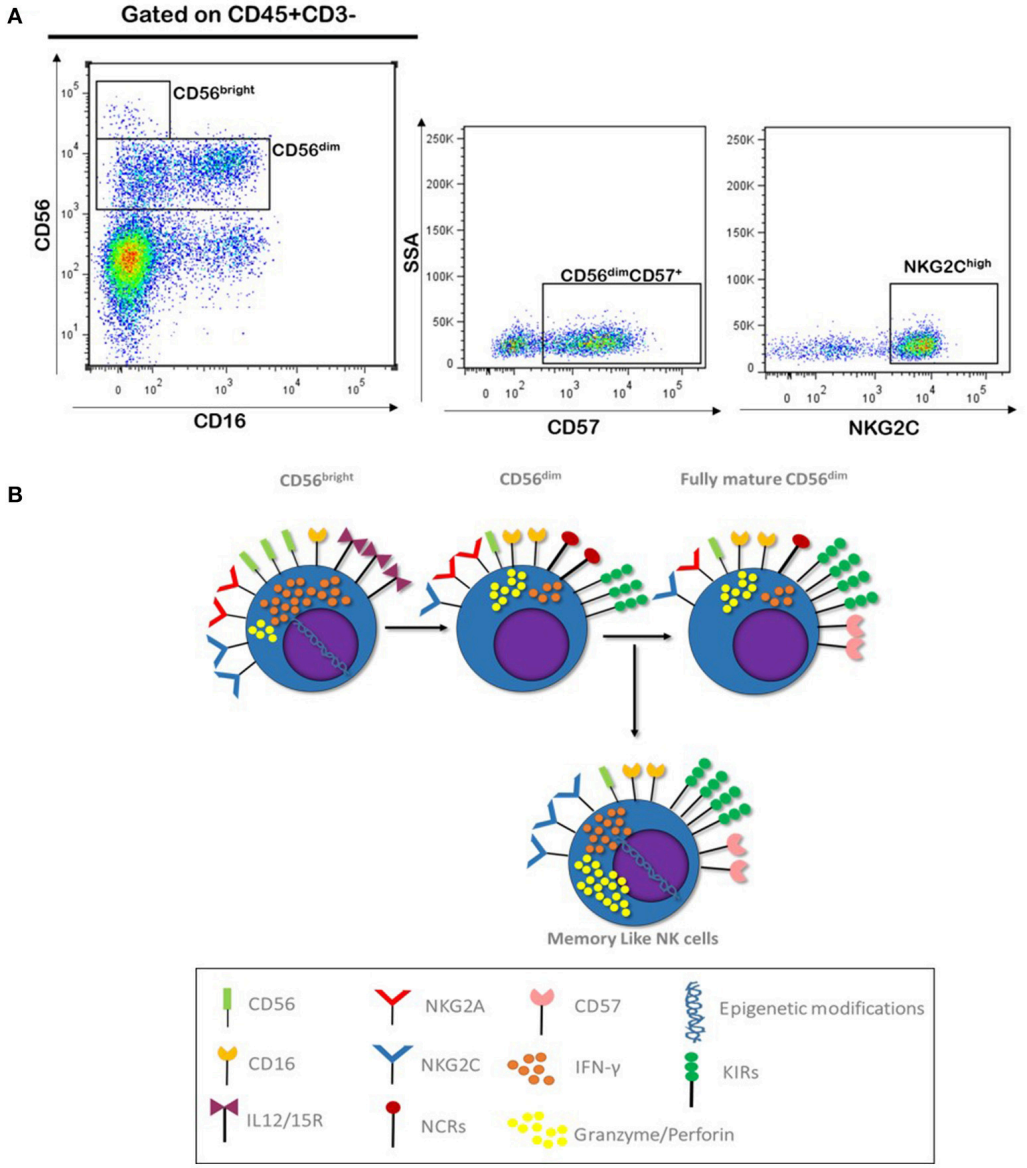
NK maturation and Memory like phenotype. **(A)** Gating strategy used for the identification of NK cells subpopulation, which includes fully mature (CD56dim CD57+) and Memory like (NKG2Chigh) NK cells; blood sample correspond to healthy donor from our laboratory (Unpublished data). **(B)** CD56^bright^ NK cells represent less mature stage of NK cells differentiation, these cells respond to IL12/15 stimulation. These also express NCRs and NKG2A/C and high affinity IL2R (CD25), which reflect their proliferative capacity. After immune licensing process, CD56^dim^ gain the expression of KIRs and loss expression of cytokines receptor, making them less reactive to cytokine stimuli, they also gain CD16 expression together with an improved cytotoxic capacity. Fully differentiated CD56^dim^ express CD57 and loss NKG2A and NCRs expression, they respond better trough KIR and NKG2C signaling. Memory like NK cells are thought to develop after CMV infection, they have a high cytotoxic capacity and present epigenetic modifications in *IFN-*γ*, TNF-*α, and *IL-10* genes allowing them to produce high amounts of these cytokines rapidly after stimuli through KIRs and NKG2C receptors.

Based on the capacity of a recall response, this subset of NK cells has been named “Memory like NK cells” and they represent a final stage of NK cells maturation. Classically, it has been thought that after CD56^bright^ differentiation to CD56^dim^, these cells retained the functional and phenotypic properties through the lifespan. However, some studies have shown that CD56^dim^ continue to differentiate; in this process, NK cells lose NKG2A expression, acquire KIR and CD57 markers, changing the expression of homing molecules like CD62L, and they display a decline in proliferative capacity and also they show epigenetic modifications in some genes like IFN-γ, TNF-α, and IL-10 (95, 96). This phenotype of NK cells has been well characterized in human cytomegalovirus (CMV) infection, where a subset of NK cells with this phenotype CD56^dim^/CD57^+^/NKG2A^−^/NKG2C^high^ is selectively expanded during acute infection and exhibit characteristics like recall response and long lived capacity (97) (Figure 2). Memory like NK cells have shown the ability to mediate adaptive immune responses to other viruses, such as vesicular stomatitis virus and HIV, and even against other pathogens such as bacteria (98, 99).

Recently an association between memory NK cells and HIV infection has been described. Reeves et al. found that a subset of NK cells from SHIV and SIV infected macaques, specifically they lysed Gag and Env-pulsed DCs in a NKG2C dependent manner, in contrast to NK cells from uninfected macaques (96). Moreover, splenic and hepatic NK cells from vaccinated macaques efficiently lysed antigen-matched DCs but not “naïve” DCs, even 5 years after vaccination. These data demonstrate that robust, durable, and antigen-specific NK cell memory can be induced in primates after both, infection and vaccination (100). HESN, exhibited higher frequency of Memory like NK cells compared with HIV infected individuals and also a positive correlation between memory NK cells and IgG titles for HCMV, which appears to be the trigger by these cells (101). Finally, HIV infected individuals showed a negative correlation between frequency of memory NK cells and viral load (102), suggesting that Memory-like subset might constitute a ready-armed immune cells able to contribute to natural control of viral load.

This evidence suggests the existence of association between the Memory like NK cells (expanded in response to CMV infection) and HIV resistance, which opens a new field in the HIV vaccine research, based in the induction of Memory like NK cells with specific responses against HIV.

## NK cell based vaccine “boosting NK cell activity”

After more than 30 years of the discovery of HIV-1, the vaccine remains elusive. Difficulties in the HIV vaccine development include: (i) integration of viral genome into the host DNA, (ii) the ability to induce immune suppression and (iii) the development of viral variants that escape from the immune control. Many attempts to design prophylactic vaccines have been focused on the induction of neutralizing antibodies that block infection by free virions; nevertheless, in the case of HIV, virus-infected cells are more infectious than free virus in both *in vitro* and *in vivo* models (103). In addition, neutralizing antibodies do not block cell to cell transmission, thus, effective design of the vaccine against HIV needs a different approach.

Accumulated evidence of the protective role of NK cells during HIV-1 infection, leads to think that boosting NK cell activity during infection or even before can help to eliminate viral reservoirs or avoid infection. The idea of boosting NK cells to cure pathologic conditions is not exclusive of HIV; in fact, a growing number of studies have been developed to enhance NK cytotoxicity as immune therapy against cancer, based on the capacity to eliminate tumor cells (104).

Increased natural cytotoxicity and ADCC mediated by NK cells have been associated with protection to HIV infection and disease progression, because those are mechanisms capable to control cell-associated virus, blocking cell to cell transmission. For this reason, the administration of molecules that can promote the activation and proliferation of NK cells have been evaluated in preclinical phase studies. For instance, it has been reported that IL-15 induce proliferation of NK and T cells in rhesus macaques (105). On 2015 Conlon et al. published the results of the first clinical trial with recombinant IL-15 in cancer patients; the administration of this cytokine resulted in increased NK cells proliferation with no toxic effects on patients and decreased of tumor size (106).

Currently, the administration of IL-15 has been considered as a part of the kick and kill strategy, which is based on the viral replication induction on latently infected cells and agents with the ability to boost NK cells activity to kill them in presence of antiretroviral therapy to avoid the infection of new cells, resulting in a diminished viral reservoir size and viral eradication in infected individuals. Garrido et al. (107) in a *ex vivo* model shown that IL-15 improves the NK cells function of healthy controls and HIV infected individuals with antiretroviral therapy, and more important these cells were able to clear latently infected cells after exposure to vorinostat (a latency reversal agent) (107).

**Figure 3:**
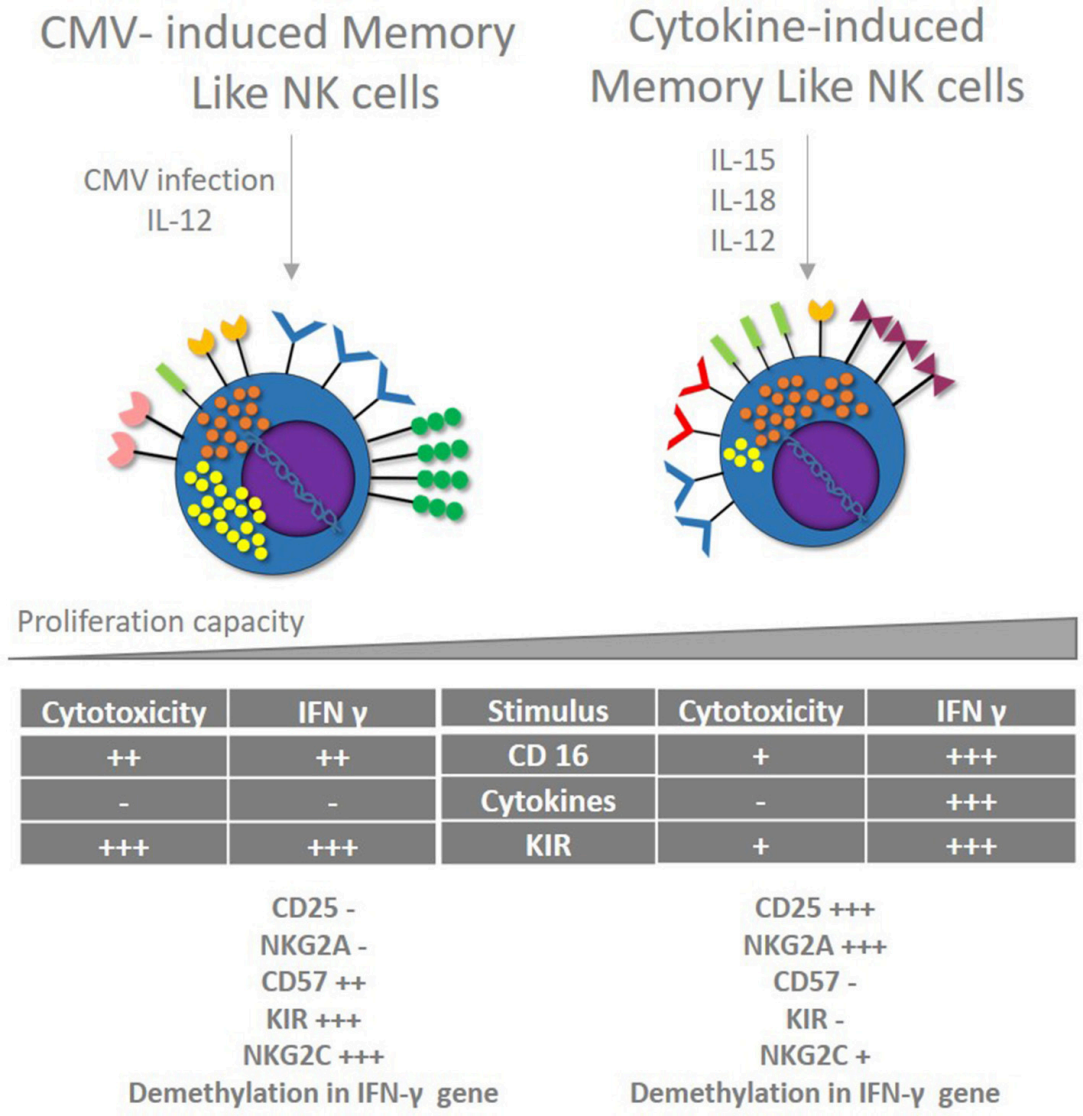
CMV and cytokines induced memory like NK cells. Memory like NK cells are developed after CMV infection in the presence of IL-12. These cells show strong cytotoxic capacity and IFN-γ production associated with epigenetic changes, restricted to certain stimulus via KIR and CD16 but no cytokines. Cytokine-induced Memory like NK cells (CIML) share some features of memory NK cells developed after CMV infection like improved IFN production secondary to epigenetic modifications; however, they show different phenotype and different cytotoxic capacity after stimulation.

Inducing Memory like NK cells will allow the development of new vaccine strategies based on cellular immunity, letting individuals to respond with strong cytotoxic activity and IFN-γ production in “specific” way during early moments of infection. Despite CMV infection seems to be the only trigger for Memory like NK cells development, cytokines cocktails induce the expansion of NK cells populations with some memory features (108) (Figure 3). Although cytokines can induce Memory like NK cells (CIML), they present a different phenotype with no expression of CD57 and KIR, and no epigenetic modifications in *IFN-*γ gene. These cells are being evaluated for cancer treatment in a clinical trial (NCT01898793) with promising results. Therefore, it is necessary to evaluate if this NK cell phenotype induced *in vitro* can be used to control HIV infection.

As mentioned before, the crosstalk between NK cells and DCs impact the establishment of both innate and adaptive immune response; in this regard, vaccine candidates based on NK-DC interaction have been proposed. Cummings et al. reported that in cocultures of NK cells with autologous DCs infected with MVA-HIV (modified vaccinia virus containing Gag, Pol, and Nef proteins from HIV), NK cells showed enhanced proliferation and modulation of cell receptor repertoire; these changes were reflected in a higher ability to control HIV-1 infection. This activity appears to be specific to HIV, as MVA-HIV-primed NK cells did not have a better ability to control other viral infections or respond against tumoral cells (109). Despite the advances in this field, none of these NK cells boosting strategies have been tested in humans.

On the other hand, the induction of antibodies that mediate ADCC has been evaluated with promising results in both preclinical and clinical studies. In fact, the evidence shows that unlike neutralizing antibodies, which are rarely induced in HIV infected individuals, ADCC-mediating antibodies are found often and in high quantity in non-progressor individuals (110). In addition, Milligan et al. reported that the levels of passively acquired ADCC-inducing antibodies were associated with decreased mortality risk in infected infants born from HIV positive mothers (111), supporting the importance of ADCC-inducing antibodies in the control of HIV infection. Additionally, Lu et al. reported that the administration of broadly neutralizing antibody accelerate the elimination of HIV-1 infected cells acting on free virus clearance and contributing to elimination of infected cells by a mechanism involving Fcγ receptors (112). Antibodies used in this studio target envelope glycoprotein gp160 and led to rapid reduction of viral load by an average 1.48 log^10^ copies/ml, this study also shown that both neutralizing and non-neutralizing antibodies can support anti HIV ADCC that was associated with both control of replication and protection against infection (112). Gómez-Román et al. showed that priming with a recombinant vaccine consisting of replication competent adenovirus with mutant SIV, followed by boosting with SIVgp120 protein, elicited the production of antibodies with ADCC activity and these antibodies were related with reduction of acute SIV viremia after a mucosal challenge of SIV in rhesus macaques (113). Recently, another type of molecule called BiKE (bi specific killer engager) has been evaluated in order to direct the NK cell-mediated ADCC against HIV-infected targets. BiKE contain the Fab portion of broadly neutralizing antibodies linked to a nanobody that binds CD16 with high affinity and induce a strong activation signal; interestingly, the use of this construct improved the ADCC and IFN-γ production capacity of NK cells in co-cultures with HIV infected cell lines (114).

Besides the success in the preclinical studies, the induction of ADCC-mediating antibodies was demonstrated to be a useful strategy for HIV-1 vaccination in human trials. The first phase III clinical trial for HIV/AIDS vaccine (AIDSVAX), which aimed to induce neutralizing antibodies, show no efficacy for prevention of acquisition or modification in HIV infection (115, 116). After this fail, AIDSVAX was evaluated in combination with another failed vaccine in the ALVAC-HIV/AIDSVAX study that used a “primed-boost” strategy. This vaccines were developed for circulating HIV subtypes from Thailand and the ADCC activity showed a significant difference in the magnitude of the response and the frequency of responders in the group of vaccines recipients compared to placebo (117). From these results, the clinical trial RV144 was design to prove the vaccine combination efficacy in other populations. In this new study, the participants lowered the rate of HIV infection by 60% at 12 months and 31.2% at 42 months compared with placebo (118). This protection was not associated with the presence of neutralizing antibodies or cytotoxic T cell responses (119); rather, it was associated with the presence of anti-HIV Env specific IgG non-neutralizing antibodies able to mediate ADCC (120). In 2011, the comparison of different immune parameters between those who received the vaccine and contracted HIV, and those who did not become infected (121), reported that subjects who produced antibodies, which recognized the V2 loop in the HIV envelope protein were 43% less likely to become infected (120).

Recently, a new variation of ALVAC-HIV/AIDSVAX has been proof in animal models with promising results. This new vaccine trial includes the recombinant poxvirus from ALVAC and the pentavalent version of AIDSVAX to increase the diversity of gp120 motifs in the antibody response. Evaluation of the antibody responses identified: (i) plasma antibody binding to HIV-infected cells, (ii) peak of ADCC antibody titers, (iii) NK cell-mediated ADCC and (iv) antibody-mediated expression of MIP-1β in NK cells; four immunological parameters with important antiviral activity as mentioned before, resulting in 55% of protection from SIV challenge (122).

In addition to strategies to boost NK cells effector function, the adoptive transfer of NK cells has been evaluated for HIV treatment. CAR expressing NK cells are modified NK cells that express chimeric antigen receptors against tumor-associated or pathogen-associated antigens redirecting the effector function to specific cells (123). This strategy has been evaluated for tumor immunotherapy, initially with T cells showing remarkable success in hematological cancers and after with NK cells on hematological cancers and solid tumors (124). In HIV context, a study by Zhen et al. (125) reported that CAR modified hematopoietic stem cells differentiate to NK cells, these cells were resistant to HIV infection and suppress viral replication *in vivo* (125); currently, the safety and tolerability of NK cells transfer is evaluated in a phase II clinical trial NCT03346499 designed to study the effects of haploidentical NK cells infusion after the stimulation with IL-2 in HIV^+^ individuals.Taken together, the evidences presented in this review highlight the role of NK cells in HIV infection; not only by the capacity to eliminate infected cells trough cytotoxic activity, but also producing soluble mediators that can block HIV entry to new cells, affecting the viral replication and spread. Beyond of these effector functions, NK cells are capable of shape the adaptive response and they are implicated in the maintenance of mucosal epithelia, a key activity to avoid the tissue damage and the subsequent microbial translocation during HIV infection. Finally, the possibility of boost the NK cells response offers a new strategy in the design of vaccines, which represent a challenge for classical vaccination and should be a priority in research to find an HIV cure.

## Author contributions

JH, WZ: Funding acquisition and Project administration. LF-Á, JH, and WZ: Writing-original draft. JH and WZ: Writing-review and editing. LF-Á, JH, and WZ: Approval of manuscript for publication.

### Conflict of interest statement

The authors declare that the research was conducted in the absence of any commercial or financial relationships that could be construed as a potential conflict of interest.
